# A method to detect influencers in social networks based on the combination of amplification factors and content creation

**DOI:** 10.1371/journal.pone.0274596

**Published:** 2022-10-06

**Authors:** Tai Huynh, Hien D. Nguyen, Ivan Zelinka, Xuan Hau Pham, Vuong T. Pham, Ali Selamat, Ondrej Krejcar

**Affiliations:** 1 Technical University of Ostrava (VŠB-TU), Ostrava, Czech Republic; 2 Kyanon Digital, Ho Chi Minh City, Vietnam; 3 Faculty of Computer Science, University of Information Technology, Ho Chi Minh City, Vietnam; 4 Vietnam National University, Ho Chi Minh City, Vietnam; 5 Faculty of Engineering–Information Technology, Quang Binh University, Dong Hoi City, Quang Binh, Vietnam; 6 Faculty of Information Technology, Sai Gon University, Ho Chi Minh City, Vietnam; 7 Malaysia Japan International Institute of Technology (MJIIT), Universiti Teknologi Malaysia (UTM), Johor Bahru, Malaysia; 8 School of Computing, Faculty of Engineering, Universiti Teknologi Malaysia & Media and Games Center of Excellence, Universiti Teknologi Malaysia, Johor Bahru, Malaysia; 9 Faculty of Informatics and Management, University of Hradec Kralove, Hradec Kralove, Czech Republic; University of Pisa, ITALY

## Abstract

A social network is one of the efficient tools for information propagation. The content is the bridge between the product and its customers. Evaluating the user’s content creation is a valuable feature to improve information spreading on the social network. This paper proposes a method for extracting brand value with influencers by combining the user’s amplification and content creation in influencer marketing. The amplification factors are studied based on the propagation of the posts on the social network in a duration time. Those factors are more valuable than before when using influencer marketing at a determined time. Moreover, the content creation score is also studied to measure content creation based on the passion point with a brand and its quality. The amplification factors and content creation score are combined to analyze posts’ interest in detecting the emerging influent users for a product in the influencer marketing campaign. Using the amplification factors, the passion points, and the content creation score, a system to manage the influencer marketing on Facebook has been constructed and tested in the real-world campaign. The experimental results show that the proposed method’s influencers bring the conversion rate’s efficiency and revenue in the influencer marketing campaign.

## Introduction

In the era of industry 4.0, a social network is a convenient tool for conveying information [1–3] and helps a brand approaching its targeted customers. Customers can find almost essential information on social networks and pay attention to the brand’s information [4, 5]. Influencer marketing is a marketing strategy that focuses on using an influencer to promote its products or services [6]. The information from an influent user gets viral diffusion on the social network [6, 7]. Besides, the brand’s information, which was propagated by the influencer, also affects the purchasing decisions of the influencer’s followers [7, 8].

In Vietnam, 65 million people are using social networks in 2019 [9], including Facebook, Tiktok, Instagram. It is 67% population of Vietnam and increased 7% comparing to in 2018 [9]. Moreover, many customers believe influencers’ reviews and an effective influencer marketing campaign can get six times the budget for that campaign [10]. Hence, determining the emerging influencers is an essential work in influencer marketing.

An influencer can be a celebrity (or macro-influencer) who has a large fan base such as celebrities, athletes, or politicians, etc., or a micro-influencer who has a small fan base but still stays a strong voice in his or her own community such as thought leaders or product reviewers, etc. According to [11], micro-influencers are defined as those who attract 1,000 to 100,000 followers and macro-influencers are user with 100,000 to 1,000,000 followers. One of the most significant differences is the fact that micro-influencers have much higher engagement rates than macro-influencers, which matters to the businesses as they can hire many micro influencers with lower costs but high possibilities of efficient marketing campaigns The engagement is a measure of how people are interacting with social media users and content. Interaction can be understood as the recipient of information has been influenced by the person giving an opinion or point of view. The term can cover a broad range of actions across all social platforms. The higher the engagement rate of the content on social networks has, the more propagating it is. This engagement or interactions is a crucial criterion for determining social media micro-influencers.

On the social network, the users’ posts determine their opinions. Therefore, there is a necessary method to analyze their posts’ sentiment value [12, 13]. In business intelligence, the brand analyzes users’ sentiment to understand what and how its customers are thinking and what their attitudes are [14, 15]. The sentiment analysis is also the foundation to measure the quality of the post’s content.

Besides amplification factors [3, 7], influencers can also create good content for attracting their audience. Thus, the evaluating of content creation helps to measure the absorption of a user’s posting. It is a valuable feature to enhance information diffusion on a social network [16, 17]. When loving a brand, a user regularly has positive posts about it, and their posts attract their audience’s interactions. An exciting post absorbs the audience if its content shows the seeder’s sentiment about a specific topic clearly [17].

**Fig 1:**
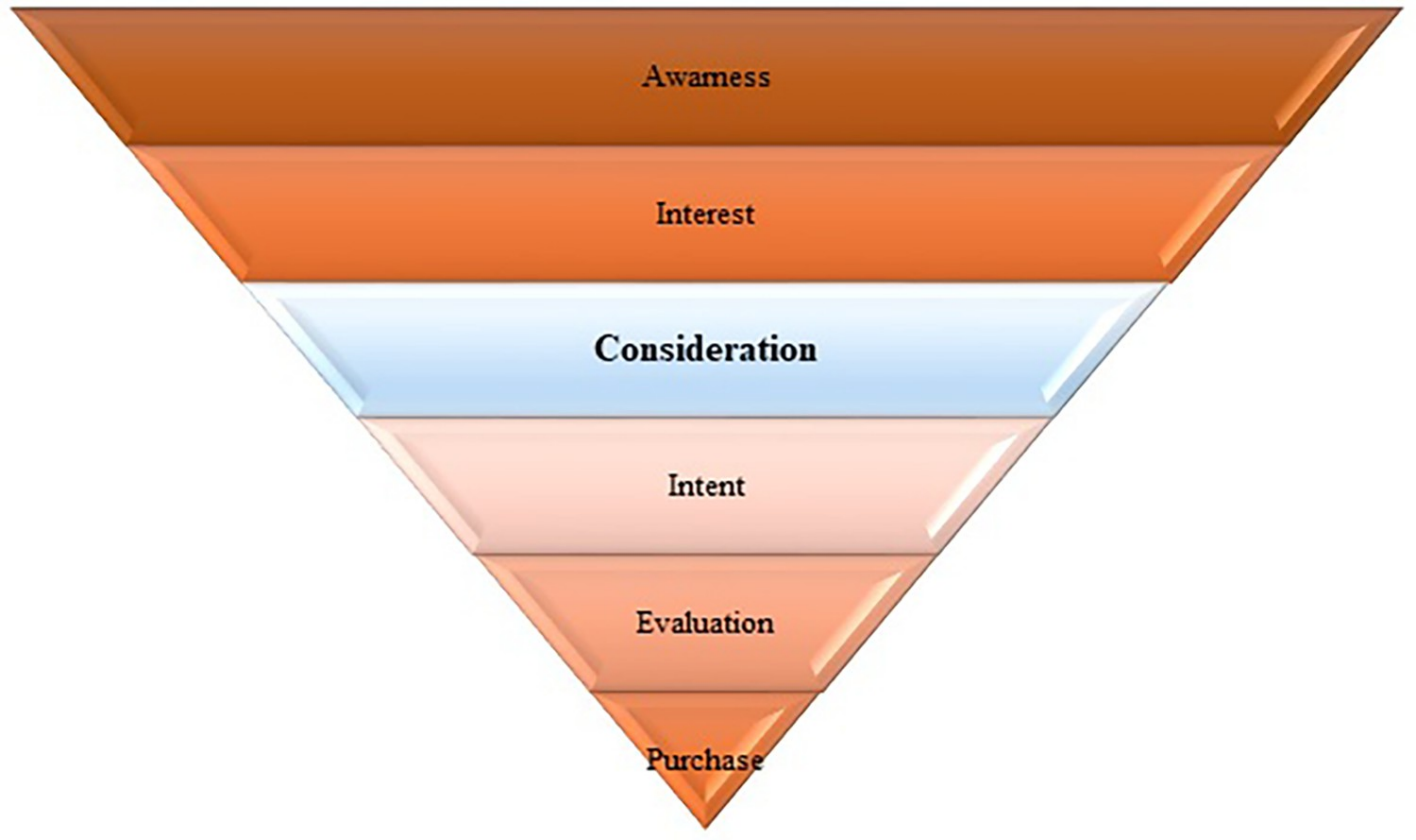
Model of purchase funnel.

Content marketing is a marketing strategy approaching targeted customers by creating valuable, relevant, and consistent content to attract the audience to drive customer action [18]. Content is the product’s information to promote customers’ purchase and plays the bridge’s role between the product and its customers. The analysis of social data content requires improving business performance in practice to achieve the expectation added value. It also engages brand awareness and develops insights into target customers [19]. In the Purchase Funnel model (Fig 1), the content impacts the consideration of a customer. The customers usually decide to order if the product’s content attracts or brings sympathy. Thus, in digital marketing, the content is essential to viral on the social network. If an influencer can create good content, his/her information will impact more people. The combination of influencer and content makes the message of a brand propagating to targeted customers effectively.

Micro-influencers usually ensure the composition of their content speaks to their audiences enough to be consistently and continuously engaged. In Fig 1, the content impacts the customer considerations. The customers commonly decide to make the order if the product’s content attracts or causes sympathy. These content acts as a bridge between the product and its customers. Or in another view, a good content strategy can create an impact on customer interactions and behaviors [20, 21]. In addition, for brand’s benefits, only content creation ability is inadequate. The content should address the influencers’ opinions, especially it will be better if such content shows the passion of influencers regarding the brand or its product and service. Positive content is a prime for massive social exposure. To express the love to a brand, a user regularly has positive posts about it, and their posts attract their audience’ interactions. An exciting post will attract the audience if its content clearly expressed the seeder’s sentiment about a specific topic. Summarize to this point, the ability to produce positive content relevance to the business, continuously and consistently, is the major criteria for micro-influencer identification. And, the micro-influencer who satisfy these two requirements are called brand advocate. Thus, we need a solution to identify the brand advocate on social networks from the influencers identification method. The real-world marketing campaign is an effort to test the proposed method for detecting influencers. It gets positive experimental results. The sale of the brand is increased, and the cost of running the marketing strategy is used more effectively.

**Fig 2:**
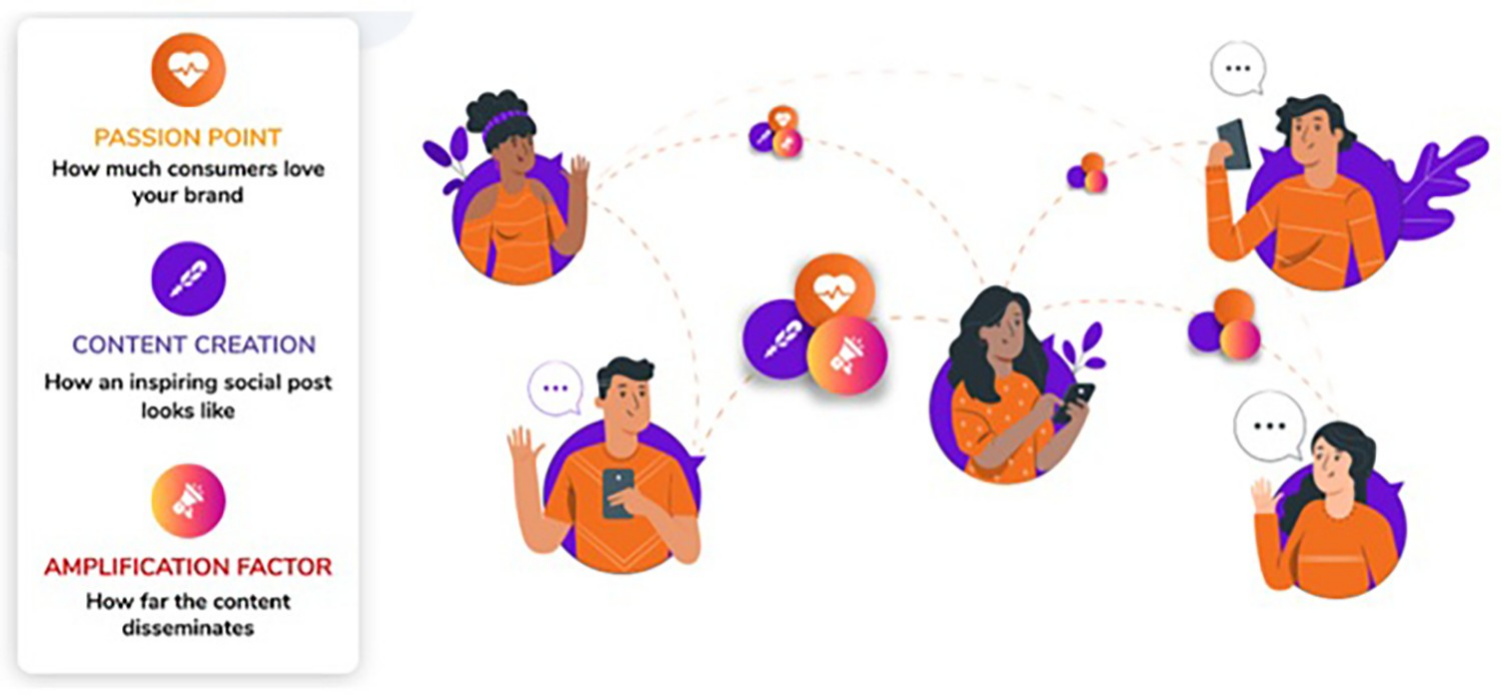
Measures of the proposed method for detecting of influencers.

This study proposes a novel approach for identifying influencer on social networks using the amplification factor to evaluate the information propagation and the content creation score to estimate user’s ability in creating contents on a social network. Firstly, a graph-base structure of a social network is introduced. This structure stores information of users and their relations to compute amplification factors on the network. Secondly, the content creation score is also studied to measure the user’s content creation based on posts’ passion and quality. The passion point is a measure to compute user favorites; it is determined based on the sentiment score of the user’s posting and his/her activity on a social network. The quality of posts is evaluated through the analysis of the content of posts. Those measures are integrated to estimate the interests of posts. Those measures are summarized in Fig 2.

The proposed method has been compared with some recent relevant methods as the baseline. It is also tested in the real world and the experiment shows that the proposed method’s influencers deliver a react-to-purchase conversion rate’s efficiency and a good return on investment in the influencer marketing campaign.

The next section of this article presents related research for detecting emerging influencers, sentiment analysis, estimating the brand’s loving of a user, and measuring content creation’s ability. Section 3 proposes some metrics to evaluate the ability of the user’s information propagation. Section 4 establishes the measures to compute the content creation score of users. Section 5 presents the method for combining the amplification factors and the content creation score to detect emerging influencers for a specific brand. The proposed method for detecting influencers based on the amplification factors and the content creation score has experimented with within reality. Section 6 shows those results. The conclusion section summarizes the main results and gives some works in the future.

## Related work

A social network is a suitable place for viral information. Although there are still fake news and negative impacts on social networks [22–24], social media is ideal for spreading positive information. It is also a popular tool to communicate and establish relationships between products/brands with their targeted customers [25]. In digital marketing, influencer marketing uses influencers to viral the information of a specific brand on the social network. Thus, enhancing opinion leaders’ affection is crucial to maximizing the influence in business [26]. If those influencers have high-quality posts, their posts will be more attractive and impact targeted audiences more effectively. Hence, identifying influencers, combining amplification factors and evaluating content creation will get essential influencers for a marketing campaign based on influencers.

The identification of prominent users in social networks s is a critical step in speeding up the spread of information, such as marketing applications, or preventing the spread of harmful content [27]. For users on a social network, the measuring of their impact on that network has been studied by many methods [22, 28], such as: using association rules [29], nomological network [30], diffusion model [31]. Those methods can be classified as Local Measures [27, 32], Short Path–Based Measures [33], Iterative Calculation–Based Measures [34, 35], Coreness-Based Measures [36], and Machine-Learning Algorithms [37, 38].

The authors in [29] proposed a method for associate learning to determine relationships between users. Those results were used to verify the identification of the most influential users. In [15, 30], some relations between value creation practices, brand community markers, and brand loyalty was built using the nomological network. This model is useful in exploring the brand’s loyal communities.

Besides, based on diffusion models’ properties in [31], influence optimization is studied. This problem’s goal is the selection of crucial opinion selecting a large part of a network. Nonetheless, those properties are general to apply to specific problems. In [39], a closeness measure to quantify users’ closeness based on interactions was defined. Incorporating this measure into the ranking mechanism is used to build an influence ranking algorithm based on PageRank, called EIRank, to evaluate our algorithm, EIRank. A dataset collected from Twitter is used to evaluate this algorithm.

Another method to recognize opinion leaders on social networks has been studied in [40], called Milestone Rank. It is the combination of selectivity measure and interest measure, which are the selection and engrossment of a user for a topic, respectively, from a set of milestones. However, Milestone Rank does not use amplification factors in a duration time.

The SNet model, which describes two main objects on the social network, such as users and posts, was proposed [31, 32]. The SNet model structure represents users’ information and actions and the relations between users and posts on a social network. In this paper, using the SNet model, the method for extracting brand value with influencers is proposed by combining the user’s amplification and content creation in influencer marketing. The amplification factors are studied based on the propagation of the posts on the social network in a duration time. Those factors are reasonable when using in the run of influencer marketing at a crucial time.

To measure the interest of a post, the attraction of its content needs to be evaluated. An exciting post will absorb many users and spread very fast on the network. It has content to determine a certain topic, and it shows the seeder’s attitude distinctly. The current methods, which evaluate a post’s content, do not have features analyzing how to write an interesting post or the user’s passion for a brand. Thus, they cannot estimate the content creation precisely to detect influencers.

The study in [27] proposed a general framework and a methodology to predict influent users who affect the behavior of other users in a time period. This method is built based on historical interactions that occurred within the online social network groups.

Sentiment analysis is the analysis of sentiments, emotions, and opinions in data [32]. It aims to evaluate the impact of news and social media [41]. The machine learning approach is an effective method for sentiment analysis [33, 42]. It also combines language-oriented to analyze the sentiment, such as self-attention neural networks and their improvement [43–45]. In [46], the relations between sentiments and the Brazilian stock market movement were constructed based on the Portuguese sentiment analysis by Multilayer Perceptron. Besides, some integrating methods of deep learning-based sentiment analysis models named lexicon were studied, combining two channels CNN–LSTM and branching of the combination CNN and LSTM/BiLSTM branches [47].

The results in [48] used a fuzzy system to design a measure of influence for an individual node in the focal network and the associated networks. The authors in [49] analyze the positive maximization influence of nodes to select the seed set with the most positive influence on the social network. However, those methods are theoretical and difficult to apply in the real-world social network. A social network includes a set of relations between objects on the network, such as users and posts. Ontology is a useful tool for representing the relationships between objects [50, 51] and building a searching system for complex information [52, 53]. Hence, with its benefits, ontology can be studied to increase the ability to detect influent nodes on the social network.

Passion point is a measure to compute the brand-loving of a user. In [54], this point is computed using some values on the users’ posts related to the specific brand. Those values are the total posts about that brand and the average reactions with each post. However, the action of the user on the social network is not mentioned in that research.

In [55], group decision-making is used to analyze discussions on a social network. In an ordinary social network discussion, a set of people disputing a certain problem can be detected by using sentiment analysis techniques. The study in [56] proposed a method to profile influential users on social media platforms. They are divided into three kinds: opinion leader, opinion reverser and topic initiator. Their profiling can reveal the difference between their opinions and dynamic evolution. The findings can support the manager to focus of attention and emotion of influencers. In the context of groups created in social networks, the research in [57] proposed a general framework and a methodology to predict influent users who impact to the behavior of other users in a time period. This method is constructed based on historical interactions that occurred within the group. Nevertheless, those methods only use to extract a set of users; they are not sufficient to retrieve the information for influencers detection.

## The proposed measures of information propagation on a social network

In this section, we describe the proposed measures of information propagation on a social network.

### Model of social network

The social network includes objects, users and posts, and relations between them [58]. Thus, the structure of this network is represented by a relational model as a graph-based. However, this model needs to be constructed the structure of a concept for representing its information completely.

**Definition 3.1 [54].** The structure of a social network is a relational model, which is a tuple **(U, P, R)**, in which, **U** is a set of users, **P** is a set of posts, and **R** is a set of relations between users and posts on this social network. This model is called the *SNet* model. The structures of each component as follows:

(1) **U**-set**:** Each *u* ∈ **U** is a user, its structure has four elements:

*u* = (*Profile*, *LPosts*, *LFriends*, *LFollowers*)

where, *Profile*: personal information of user *u*.

*LPosts* = [*p*_1_, *p*_2_, …, *p*_*n*_]: List of posts *p*_*i*_ ∈ **P**, which are related to user *u* (*i* = 1…*n*)

*LFriends* = [*f*_1_, *f*_2_, …, *f*_*m*_]: List of other user *f*_*j*_ ∈ **U**, which are friends of user *u* (*j* = 1…*m*).

*LFollowers* = [*l*_1_, *l*_2_, …, *l*_*q*_]: List of other user *l*_*k*_ ∈ **U**, which are followers of user *u* (*k* = 1…*q*).

(2) **P**-set**:** Each *p* ∈ **P** is a post, it includes six elements:

*p* = (*Content*, *Seeder*, *τ*, *Reaction*, *Sh*, *Com*)

where, *Content*: the content of post *p*.

*Seeder* ∈ **U**: this is the user as the seeder of post *p*.

*τ* ∈ TIME: the timestamp of post *p* (Time is the data type as timestamp).

*Reaction*: the set of users who reacted with post *p*.

*Sh*: the set of users who shared with post *p*.

*Com*: set of users who has comments on *post p*.

The structures of *Reaction*, *Sh*, and *Com* sets are defined in Def. 3.2.

(3) **R**-set**:** Each relation in **R** is one of two kinds:

$$R=R_{U}\cup R_{P}$$

where, **R**_**U**_: a set of relations between two users. the content of post *p*. It includes:

+ friend ⊆ **U** × **U**: a user is a friend of another user.

+ follower ⊆ **U** × **U**: a user is a friend of another user.

**R**_**P**_**:** a set of relations between a user and a post. It includes:

+ comment ⊆ **U** × **P**: a user comments on a post.

+ share ⊆ **U** × **P**: a user shares a post.

+ reaction ⊆ **U** × **P** × **N**: a user reacts to a post. Each kind of a reaction is a natural number.

**Definition 3.2.** Given a post *p* ∈ **P**, the structures of *p*.*Sh*, *p*.*Com* and *p*.*Interaction* are organized as follows:

*p*.*Sh*: = {(*v*, π_*v*_) ∈ **U** × Time | share(*v*, *p*), π_*v*_ ∈ Time is the timestamp of the user *v* shares the post *p*}

*p*.*Com*: = {(*v*, π_*v*_) ∈ **U** × Time | comment(*v*, *p*), π_*v*_ ∈ Time is the timestamp of the user *v* comments on the post *p*}

*p*.*Reaction*: = {(*v*, π_*v*_, *s*) ∈ **U** × Time × N | reaction(*v*, *p*, *s*), π_*v*_ ∈ Time is the timestamp of the user *v* who reacts on the post *p*, *s* is the kind of this reaction}

In which, Time is the data type as timestamp.

### Amplification factors of a user

**Definition 3.3.** (the reaction point). Given a post *p* ∈ **P**.

a/ The point of a reaction of a user *u* for the post *p*, point_*p*_(*u*), is a map:

          point_*p*_: **U** → [0, 1]

$${\mathrm{point}}_{p}(u)≔\{\begin{array}{ll} 0 & \mathrm{if}\;\mathrm{not}\;\mathrm{reaction}(u,p,s)\\ f(s) & \mathrm{if}\;\mathrm{reaction}(u,p,s) \end{array}$$

where, *f*: N→ [0,1] is an evaluation function for each kind of reactions.

b/ The point of reactions for the post *p*, react_point(*p*), is computed as followed:

$$\mathrm{react}\_\mathrm{point}(p)=\frac{\gamma_{1}*\sum_{v\in I_{1}(p.Seeder)}{\mathrm{point}}_{p}(v)+\gamma_{2}*\sum_{v\in I_{2}(p.Seeder)}{\mathrm{point}}_{p}(v)+\gamma_{3}*\sum_{v\in I_{3}(p.Seeder)}{\mathrm{point}}_{p}(v)}{\mathrm{card}(p.\mathrm{Reaction})}$$

where, $I(p)≔\underset{u\in p.\mathrm{Reaction}}{\cup }u$: set of users reacting on the post *p*.

*I*_1_(*p*): = {*u* | *u* ∈ *I*(*p*) and friend(*u*, *p*.*Seeder*)}: set of users who are friends of the user *p*.*Seeder*, and they react on the post *p*.

*I*_2_(*p*): = {*u* | *u* ∈ *I*(*p*) and follower(*u*, *p*.*Seeder*)}: set of users who are followers of the user *p*.*Seeder*, and they react on the post *p*.

*I*_3_(*p*): = *I*(*p*) \ (*I*_1_(*p*) ∪ *I*_2_(*p*)): set of users who are unrelated to the user *p*.*Seeder*, and they react on the post.

*γ*_1_, *γ*_2_, *γ*_3_: are weighted numbers, 0 < *γ*_1_ ≤ *γ*_2_ ≤ *γ*_3_ < 1. The detection of those weighted numbers is based on the characteristic of social network.

**Definition 3.4**. (The metrics of a user) Let F = (**U, P, R**) be a social network as SNet model, and user *u* ∈ **U**.

a/ Some metrics of the user *u* are shown in Table 1.

**Table 1:** Metrics of a user.

Measures	Meaning	Formulas
*SI*(*u*)	compute the effect of the user’s post in terms of the shared posts.	$SI(u)=\frac{\alpha_{1}.\mathrm{card}(SU_{1}(u))+\alpha_{2}.\mathrm{card}(SU_{2}(u))+\alpha_{3}.\mathrm{card}(SU_{3}(u))}{\mathrm{card}(F_{u})}$
*CI*(*u*)	compute the impact of comments on *u*’s posts.	$CI(u)=\frac{\beta_{1}.\mathrm{card}(CU_{1}(u))+\beta_{2}.\mathrm{card}(CU_{2}(u))+\beta_{3}.\mathrm{card}(CU_{3}(u))}{\mathrm{card}(F_{u})}$
*Ir*(*u*)	The reaction ratio with the post of the user *u*.	$Ir(u)=\frac{\sum_{p\in u.ListPosts}\mathrm{react}\_\mathrm{point}(p)}{\mathrm{card}(u.ListPosts)}$
*Imp*(*u*)	the average impact of sharing, commenting, and interacting of user *u*.	$\mathrm{Imp}(u)=\frac{\alpha .SI(u)+\beta .CI(u)+\gamma Ir(u)}{\alpha +\beta +\gamma }$
*Popularity* (*u*)	popularity measure of a user.	$Popularity(u)=1-e^{-\lambda .card(F_{u})}$

where, *SU*_1_(*u*) (*SU*_2_(*u*) and *SU*_3_(*u*)) is the set of users who share *u*’s posts, and those users are friends (followers and unrelated users) of the user *u* (resp.)

*CU*_1_(*u*) (*CU*_2_(*u*) and *CU*_3_(*u*)) is the set of users who comment on *u*’s posts, and those users are friends (followers and unrelated users) of the user *u* (resp.)

α_1_, α_2_, α_3_, β_1_, β_2_, β_3_: are weighted numbers. 0 < α_i_, β_j_ < 1 (1 ≤ *i*, *j* ≤ 3)

α, β, *γ*: are weighted numbers. 0 < α, β, *γ* < 1

*F*_*u*_: = *u*.*ListFriends*, ∪ *u*.*ListFollowers*, and λ: constant.

b/ The *influential vector* measures the influence of the user *u* is as follows:

IU(u)≔(Imp(u),Popularity(u)) (1)

The formula of IU(*u*) as a vector is similar to [36]. However, the determination of each element, *Imp*(*u*) and *Popularity*(*u*), is improved.

Some conditions:

- An unrelated user is only concerned about the post if this post is inspiring and attractive on the social network, so the weight for unrelated users’ reactions is higher than the weight for others’ reactions. A friend is usually more excited than a follower, so the weight for friends’ reactions is lower than the weight for the reactions of followers [17, 18]. Thus, we have conditions: α_1_ ≤ α_2_ ≤ α_3_ and β_1_ ≤ β_2_ ≤ β_3_.
- When a post is shared, the user thinks this post was useful to others; when a post is exciting, the user comments on it; the “like”-pressing may be a habit [17, 18]. Thus, we have the condition: 0 < *γ* ≤ β ≤ α < 1.

**Definition 3.5**. Given a user, *u* ∈ **U**, a post *p* ∈ **P**, and the time window δ. The set of users interacting with the post *p* of user *u* in the time window δ is:

$$\begin{array}{l} I_{p}^{u}(\delta )=\{user\in U|(user,\pi_{user})\in U\times \mathrm{TIME},\\ \;user\in (p.\mathrm{Reaction}\cup p.Sh\cup p.Com),\\ \;user\neq u,\pi_{user}\in [p.\tau ,p.\tau +\delta ]\\ \;\} \end{array}$$ (2)

where π_*user*_ is the timestamp when the user reacts, shares, or comments on the post *p*.

### Content creation score

The post’s content is very significant to attract audiences engaging in its information. In this section, a measure for estimating the quality of content creation is proposed. This measure is established by the combination of sentiment score and passion point [59, 60]. The method in this section was improved from results in [59].

#### Sentiment score

Sentiment analysis is the classification of human emotions by using techniques of text analysis. The sentiment score measures a personal person’s feelings about a specific brand by analyzing words which were used to debate or discuss it. In this section, the sentiment of posts on a social network is analyzed by the sentiment lexicon. The attributes of positivity and negativity are utilized to evaluate the sentiment score of a post.

**Definition 4.1 [59]:** The *sentiment score* of a word *ω*, denoted *SS*(*ω*), is determined as:

SS(ω)≔PI(ω,posi)−PI(ω,nega) (3)

where *posi* (and *nega*) is the positive (and negative) content. The function *PI*, which indicates the pointwise mutual information, is computed by followed formulas:

$$PI(\omega ,posi)≔\log\frac{fr(\omega ,posi).T}{fr(\omega ).fr(posi)}$$ (4)

$$PI(\omega ,nega)≔\log\frac{fr(\omega ,nega).T}{fr(\omega ).fr(nega)}$$ (5)

where *fr*(*ω*, *posi*) (and *fr* (*ω*, *neg*)) is the frequency of word *ω* appearing in positive (and negative) posts (resp.), *fr*(*ω*) is the frequency of the word *ω* in total posts of the corpus, and *T* is total posts. We noted that all posts in the corpus were labeled positive or negative content.

Thus, from (3)(4)(5), we have:

$$SS(\omega )≔\log\frac{fr(\omega ,posi).fr(nega)}{fr(\omega ,nega).fr(posi)}$$ (6)

**Definition 4.2:** Given a post *p* = *ω*_o_*ω*_1_*ω*_2_…*ω*_*m*_, where *ω*_*j*_ is a word (0 ≤ *j* ≤ *m*). The *sentiment score* of post *p*, denoted *SS*(*p*), is computed by the followed formula:

$$SS(p)≔\sum_{k=0}^{m}SS(\omega_{k})$$ (7)

**Definition 4.3:** Given a post *p*, *φ* > 0 is a constant.

- The post *p* is negative if and only if *SS*(*p*) ≤ -*φ*.
- The post *p* is neutral if and only if -*φ < SS*(*p*) < *φ*.
- The post *p* is positive if and only if *SS*(*p*) ≥ *φ*.

#### The formula to compute passion point

The measure of the user’s loving of a brand is called Passion point. In [54], this point’s formula is computed by the Wilson score interval method for the binomial proportion confidence interval [61].

**Definition 4.4 [54]:** Let *u* ∈ **U** be a user, a brand *X*.

a) The ranking score of the user *u* with brand *X*:

$${\mathrm{rank}}_{X}(u)≔\frac{\rho +\frac{z^{2}}{2n_{u}}}{1+\frac{z^{2}}{n_{u}}}-\frac{z}{1+\frac{z^{2}}{n_{u}}}\sqrt{\frac{\rho (1-\rho )}{n_{u}}+\frac{z^{2}}{4n_{u}^{2}}}$$ (8)

where *n*_*u*_ = the number of posts of the user *u*,

*n*_*X_positive*_ = the number of positive posts of the user *u* with the brand *X*.


$$\rho =\frac{n_{X\_positive}}{n_{u}}:\;\mathrm{the}\;\mathrm{binomial}\;\mathrm{proportion}$$

*z*: the quantile of a normal distribution.

b) The formula computes the passion point of the user *u* with brand *X* [54]:

$$oldPP_{X}(u)≔rank_{X}(u)+log(n_{u})$$ (9)

However, the activeness of the user is not mentioned in the Formula (9). In practice, the more a user is interested in the brand, the more he/she has activities related to it. For example, if a certain person loves the brand, he/she will frequently dedicate and contribute to this brand on social media platforms. Hence, a user is more active with a brand; he/she is more passionate, dedication, and contribution to increase the brand value on the social network. The Formula (9) is improved by combining the feature of activities.

**Definition 4.5:** (Passion point)

Let *u* ∈ **U** be a user, and a brand *X*.

a) The activeness of the user *u* with the brand *X* is computed by:

$$Active_{X}(u)≔\frac{n_{X}{}_{\_positive}}{n_{day}}$$ (10)

where, *n*_*day*_ = the number of report days.

b) The passion point, denoted *PP*_*X*_(*u*), is computed by:

$$PP_{X}(u)≔Active_{X}(u)*oldPP_{X}(u)$$ (11)

#### The quality of posts

Given a social network F = **(U, P, R)** as SNet model, and a user *u* ∈ **U**, a post *p* ∈ **P** on the social network F. Denote:

- *word*(*p*): the quantity of words in the post *p*.
- *word*_*pos*_(*p*): the quantity of positive words in the post *p*.

The method for estimating the content quality of the user’s posts is proposed in this section. In common practice, the posts which are too short cannot give full information, especially the information about products. They are not useful for influencers to attract their audience by introducing a product. In this study, the posts with a small word are considered as meaningless In this study, the posts with a small word are considered as meaningless in advertising, they must have an appropriate length. Hence, only meaningful posts are considered when evaluating the content quality of the user’s posts. In this study, a meaningless post is a post whose words are smaller than the average quantity of words in each post. After excluding meaningless posts, the content quality of posts is determined based on the remaining posts.

The content quality of *u*’s posts, denoted *Q*(*u*), is estimated as follows:

**Step 1:** Ascending sorting of posts in *u*.*ListPosts* by their number of words.

Step 2:

- Let *k*: = ⌊σ.*card*(*u*.*ListPosts*)⌋, where σ is a constant, 0 < σ < 0.5, and ⌊*y*⌋ is the greatest integer less than or equal to *y*.
- Select *k* posts in *u*.*ListPosts* which have the least number of words.
- Determine:

$$\phi ≔\frac{\sum_{\begin{array}{c} i=1\\ p_{i}\in u.ListPosts \end{array}}^{k}word(p_{i})}{k}$$ (12)

**Step 3:** The quality of posts for user *u* is estimated by:

$$Q(u)≔\frac{\sum_{\begin{array}{c} p\in u.ListPosts\\ w(p)\geq \phi \end{array}}\frac{word_{pos}(p)}{word(p)}}{card(\left\{p\in u.ListPosts|word(p)\geq \phi \right\})}$$ (13)

#### Content creation score

When a user loves a brand, he/she will create some high-quality, attractive posts on a social media platform to acquaint his/her audience with that brand [4, 17]. The content creation score estimates a user’s ability to attract an audience through his/her post. For a user *u*, this score is computed by combining of the passion point with a brand *X*, *PP*_*X*_(*u*), and the quality of posts’ content, *Q*(*u*).

**Definition 4.6:** (Content creation score)

Let *u* ∈ **U** be a user and a brand *X*. The content creation scores of the user *u* for the brand *X*, denoted *CC*_*X*_(*u*), is computed as follows:

$$CC_{X}(u)≔PP_{X}(u)+\log(Q(u))$$ (14)

In which, *PP*_*X*_(*u*) and *Q*(*u*) are determined by (11) and (13), resp.

The Eq (14) determines the content creation score by combining the posts’ passion and content quality. The value of *PP*_*X*_(*u*) will increase when the user is passionate about the brand *X*, so the user will create some high-quality posts to introduce that brand. In the practice, there are users who regularly posting positive contents to a brand, but those contents are nevertheless the same. Besides, the passion point is a user cumulative score on the brand which will be will accumulated gradually through the time of interaction and sharing of information; then, we can underestimate the creativity of these users [21]. The value of *Q*(*u*) in (14) performs the quality of posts through positive words. If users have a low content creation, although they use many positive words, those words will be repeated many times. Thus, the role of log(*Q(u)*) in (14) will omit those repeated positive words in posts.

## The combination method for detect influencers on a social network based on content creation

Homophily and social reinforcement are two characteristics of community structure on a social network. Homophily states that comparable individuals engage and share content more frequently than other users [62]. Indeed, users are more likely to bond with those who share similar interests, and various studies have demonstrated that homophily among users has an impact on the predictability of user profiles [63] and that it may be effectively used for link prediction and product suggestion [64]. Social reinforcement is the behavior of one person, which can affect other people who have relations with him/her, such as his/her audiences or friends/followers of audiences. This section proposes a method for detecting emerging influencers of a given product or brand based on the combination of information propagation and content creation score.

### Create the homophily of a determined brand

Homophily means that similar individuals associate with each other more often than others on social networks [65]. Instant advertising and massively targeted advertising both employ the homophily notion to understand how a user’s friends influence the predictability of his or her behavior or to promote things. Homophily can be observed in online social networks, but there is difficult to analysis investigate the principle of homophily. The results in [66] show that a simple product of degree and homophily measures can be quite effective in guiding local search. This section presents a method to construct a sub-graph showing a group of users who are fond of the determined brand as homophily. This analyzing uses the passion point and content creation score to evaluate users in social network.

#### Algorithm for creating of the homophily of a determined brand

**Definition 5.1 [32].** Let F = (**U, P, R**) be a social network as SNet model.

The weighted graph *G* = **(V, E)** contains the links between users on the network F, in which **V** is a set of vertexes representing users in **U**, and **E** is a set of weighted edges representing the relations between users. The computing of the weight for each edge *e* ∈ **E**, denoted *w*(*e*), is shown as follows:

If follower(*u*_*i*_, *u*_*j*_), then *w*(*e*_*ij*_) = 1.

If friends(*u*_*i*_, *u*_*j*_), then *w*(*e*_*ij*_) = *w*(*e*_*ji*_) = 2.

For *p* ∈ **P** and *u*_*k*_ = *p*.*Seeder*:

For each *u*_*i*_ ∈ **U** and *u*_*i*_ ≠ *u*_*k*_ do:

- If reaction(*u*_*i*_, *p*, *s*), then *w*(*e*_*ik*_) + = 1.
- If comment (*u*_*i*_, *p*), then *w*(*e*_*ik*_) + = 2.
- If shared (*u*_*i*_, *p*), then *w*(*e*_*ik*_) + = 1.

In this section, a method for building a sub-graph of the graph representing the social network is proposed based on a given brand or product. This method will extract a sub-graph showing a group of users who are fond of the brand. That sub-graph can detect the homophily for the given brand.

  **Algorithm 1:** Construct a sub-graph showing a group of users who are fond the brand.

    **Input:** The specific brand *X*.

        Graph *G* represents the relations between users on social network F = **(U, P, R)**.

    **Output:** A sub-graph of users loving brand *X*.

The followed algorithm presents the constructing of the sub-graph:

  **Step 1:** For each user *u* ∈ **V** of the graph *G*.

    Let a constant ω > 0 be the minimum value of the passion point for the brand *X*.

    Check *u*.*ListPost*s. If the user *u* mentioned to brand *X* in his/her posts.

    **If** *PP*_*X*_(*u*) ≥ ω, where *PP*_*X*_(*u*) is determined by the Eq (11):

        **Add** the node *u* into the sub-graph;

        **Goto** Step 2;

  **Step 2:** Extend to neighbors of the current node.

    **Add** an edge between the current node *u*, and it is neighbor *v* into the sub-graph if:

    Case 1: The neighbor *v* also mentioned to the brand *X*.

      • Create an edge between user *u* and the neighbor *v* with its weight determined as Definition 5.1.

     • If the post *p* of the user *v* is related to the brand *X* and that post is shared from a user *y* = *p*.*Seeder* (*y* ≠ *v*), make an edge between this neighbor *v* and the user *y*.

    Case 2: The neighbor *v* interacts or comments on *u*’s posts related to *X*.

  **Step 3: If** there are still nodes that have not yet been traversed in the network

          **Goto** Step 1.

#### The complexity of Algorithm 1

When considering users on a social network, the have to adequately numbers of friends, followers and posts on that network. In this section, the algorithm 1 will be estimated its complexity based on those parameters in the assuming that all users have the same about the number of posts, the number of friends and the number of followers.

Given a social network F = **(U, P, R)** as SNet model, and a brand *X*. Denote:

- *n* = card(**U**): number of users on the network,
- *m*: the average number of posts for each user.
- *u*_1_: the average number of friends for each user.
- *u*_2_: the average number of followers for each user.
- *L*_*X*_: List of keywords related to the brand *X*.

**Lemma:** Given a post *p* ∈**P**, and a brand *X*. The complexity for determining the post *p* related to the brand *X* is:

$$O(\mathrm{card}(L_{X}).word(p))$$ (15)

where, *word*(*p*) is the number of words in the post *p*.

**Theorem 1:** The complexity of the algorithm 1 is:

$$O(\omega .\mathrm{card}(L_{X}).n^{2}.m)$$ (16)

where, *ω* is the average number of words for each post.

* Proof: There are two main steps in Algorithm 1: Step 1 and Step 2.

+ Step 1 of the algorithm 1:

For each user *u* ∈ **U**, we need to do:

- Step 1.1: Determine the user *u* mentioned the brand *X* in his/her posts or not.
- Step 1.2: Compute *PP*_*X*_(*u*) by the Eq (11).

From Lemma, the complexity of step 1.1 is as follows:

$$\begin{array}{l} \;O\left(\sum_{p\in u.ListPosts}\mathrm{card}(L_{X}).word(p)\right)\\ =O\left(\mathrm{card}(L_{X}).\sum_{p\in u.ListPosts}word(p)\right) \end{array}$$ (17)

We have:

$$\sum_{p\in u.ListPosts}word(p)\approx \frac{\sum_{p\in u.ListPosts}word(p)}{m}.m,$$

where *m* is the average number of posts for each user.

= *ω*.*m*, *ω* is the average number of words for each post.

Thus, the Formula (17) can be written as follows:

$$O(\mathrm{card}(L_{X}).\sum_{p\in u.ListPosts}word(p))\approx O(\mathrm{card}(L_{X}).\omega .m)$$ (18)

At the step 1.2, by the Formula (11), the complexity of computing of

$$PP_{X}(u)\;is:\;O(n)$$ (19)

Hence, because card(**U**) = *n*, and from (18)(19), the complexity of Step 1 of Algorithm 1 is:

$$\begin{array}{r} O(\max(n.\mathrm{card}(L_{X}).\omega .m,n^{2}))=O(n.\mathrm{card}(L_{X}).\omega .m)\\ =O(\omega .\mathrm{card}(L_{X}).n.m) \end{array}$$ (20)

+ Step 2 of the algorithm 1:

For each user *u* ∈ **U** and a user *v* ∈ *u*.*ListFriends* ∪ *u*.*ListFollowers*, we have two cases:

- Case 1: The user *v* mentioned the brand *X* in a post *p*.

+ Create an edge between vertexes *u* and *v*.

+ If the post *p* is shared from a post of the user *y* = *p*.*Seeder*, create an edge between vertexes *v* and *y*.

From Lemma, the numbers of friends, followers and posts of user *u* is *u*_1_, *u*_2_, and *m* respectively, we have the complexity of Case 1 is:

$$O(\omega .\mathrm{card}(L_{X}).(u_{1}+u_{2}).m)$$ (21)

- Case 2: Identify the interaction of the user *v* on user *u*’s posts which are related to the brand *X*.

From Lemma 1, the numbers of friends, followers and posts of user *u* is *u*_1_, *u*_2_, and *m* respectively, we have the complexity of Case 2 is:

$$O((u_{1}+u_{2}).m)$$ (22)

By (21) and (22), card(**U**) = *n*, the complexity of Step 2 is:

$$O(\max(n.\omega .\mathrm{card}(L_{X}).(u_{1}+u_{2}).m,n.(u_{1}+u_{2}).m))=O(\omega .\mathrm{card}(L_{X}).n.(u_{1}+u_{2}).m)$$ (23)

(because *ω* > 1 and $\frac{card(L_{X})}{n_{L_{X}}}>1$)

+ The complexity of Algorithm 1:

From (20) and (23), the complexity of Algorithm 1 is as follows:

$$\begin{array}{l} O(\max(\omega .\mathrm{card}(L_{X}).n.m,\omega .\mathrm{card}(L_{X}).n.(u_{1}+u_{2}).m))\\ =O(\omega .\mathrm{card}(L_{X}).\max(n.m,n.(u_{1}+u_{2}).m))\\ =O(\mathrm{card}(L_{X}).n.(u_{1}+u_{2}).m.\omega ) \end{array}$$ (24)

We have *u*_1_ and *u*_2_ are the average numbers of friends and followers of a user, so: *u*_1_ ≤ *n* and *u*_2_ ≤ *n*. From (24), the complexity of Algorithm 1 is:

$$\begin{array}{l} O(\mathrm{card}(L_{X}).n.(u_{1}+u_{2}).m.\omega )\\ =O(\mathrm{card}(L_{X}).n.2n.m.\omega )\\ =O(\mathrm{card}(L_{X}).n^{2}.m.\omega )\;(q.e.d) \end{array}$$ (25)

In practice, with a determined business sector, the list *L*_*X*_ is a set of featured keywords for the brand *X*. Thus, marketing experts in that sector will determine the list *L*_*X*_. Hence, by (25), the complexity of Algorithm 1 is: *O*(*n*^2^.*m*.*ω*)

### The influencers based on the content creation propagation

Content creation propagation on the posts has been represented by user influence and the number of successful propagations based on computing the user’s post’s quality and the user’s passion point.

**Definition 5.2:** Given a user *u* ∈ **U**, a post *p* ∈ **P**, the time window δ, and the brand *X*. The user *u* is the seeder of *p*, *u* = *p*.*Seeder*, and the post *p* is related to brand *X*.

A set of users, who propagate the content *p* in the time window δ with the determined threshold of content creation scores, is determined as follows:

$$IPCC_{p}^{X}(\delta )≔I_{p}^{u}(\delta )\cap p.Sh\cap \{v\in U|CC_{X}(v)\geq \theta \}$$ (26)

where, θ is the threshold of content creation score,

$I_{p}^{u}(\delta )\;\mathrm{and}\;CC_{X}(v)$ are computed by the Eqs (2) and (14), resp.

**Definition 5.3**: Given a user *u* ∈ **U**, a post *p* ∈ **P**, the time window δ, and the brand *X*.

a/ The social pulse of the post *p* for the brand *X* in the time window δ is the value:

$$SP_{p}^{X}(\delta )=\sum_{v\in IPCC_{p}^{X}(\delta )}\mathrm{card}(I_{p}^{v}(\delta ))$$ (27)

b/ The average of interactions based on the content creation for *u*’s posts related to the brand *X* in the time window δ is:

$$AICC_{u}^{X}(\delta )≔\frac{\sum_{\begin{array}{l} p\in u.ListPosts\\ p\mathrm{is}\;\mathrm{related}\;\mathrm{to}\;X \end{array}}SP_{p}^{X}(\delta )}{\mathrm{card}(u.ListPosts)}$$ (28)

By the Eq (1), the measure of the impact of the user *u* based on a 2D vector of amplification factors: IU(*u*): = (*Imp*(*u*), *Popularity*(*u*)). Because of directly showing the affection of the user *u* on the social network, the value of *Imp*(*u*) needs to be a priority when comparing the influence between two users. The lexical order between two vectors is reminded in Definition 5.4.

**Definition 5.4:** (The lexical order)

Let ∇ be a set of real value, and vectors a = (a_1_, a_2_) ∈ ∇^2^, and b = (b_1_, b_2_) ∈ ∇^2^. Define:

$$a\leq b\Leftrightarrow [\begin{array}{l} a_{1}<b_{1}\\ a_{1}=b_{1}\;\mathrm{and}\;a_{2}\leq b_{2} \end{array}$$

**Definition 5.5** (influential user/influencer).

a/ The user *u* is more influent than the user *v* in the time window δ, denoted *v* $\bar{x}$*u*, if:

$$\begin{array}{l} i.\;IU(v)\leq IU(u)\;\mathrm{and}\;AICC_{v}^{X}(\delta )\leq AICC_{u}^{X}(\delta )\\ \mathrm{ii}.\;\mathrm{OR}\;(Popularity(v),AICC_{v}^{X}(\delta ))\leq (Popularity(u),AICC_{u}^{X}(\delta )) \end{array}$$

b/ Let a group of users *G* ⊆ **U**, a user *w* ∈ *G* is an *influential user* on F in the time window δ for the brand *X* if:

$$\mathrm{card}(\left\{v\in G|v\ll_{X}u\right\})\geq \mu \times \mathrm{card}(G)$$ (29)

where *μ* is a constant, 0 < *μ* < 1.

### Determining of the Influencers on a social network combining the content creation score

#### Algorithm for determining of the Influencers on a social network

For a given brand, the influencers on the social network can convey the brand’s information to target audiences by using the passion point and content creation score. The process for determining those influencers is as the followed algorithm:

Let F = (**U, P, R**) be a social network as the SNet model and a brand *X*. Algorithm 2 detects the brand *X’s* potential, influential users, who can be selected to run a campaign of influencer marketing on the social network F in the time window δ. Those influencers also can create excellent content to attract their audiences.

**Algorithm 2:** Determine the emerging influencers.

• **Stage 1:** Determine homophily being a group of lovers of brand *X*.

    Step 1: Create a graph *G* representing relations between users on social network F as Definition 5.1.

    Step 2: Using Algorithm 1, construct a sub-graph of *G* to determine homophily who love the brand *X*.

        This group is denoted *G*_*X*_.

• **Stage 2:** Detect the influencers combining the evaluation of their content creation.

    Step 3: For each user *u* ∈ *G*_*X*_, compute the influent metrics of the user *u*.

        • Influent vector IU(*u*): = (*Imp*(*u*), *Popularity*(*u*)) as the Formula (1).

        • The content creation score CC_*X*_(*u*) as Formula (14).

        • The average of interactions based on the content creation for *u*’s posts related to the brand *X*: $AICC_{u}^{X}(\delta )$, is calculated by the Formula (27).

    Step 4: Detect the set of emerging influencers in *G*_*X*_ as Definition 5.5.

        *S*: = {};

        **for** each user *u* in *G*_*X*_ **do**

        {

                *S*_*u*_(δ): = {*w* ∈ *G*_*X*_ | *w* $\bar{x}$*u*}, in which, the relation “$\bar{x}$” was defined as Definition 5.5.

                **If** card(*S*_*u*_(δ)) ≥ *μ* × card(*G*_*X*_) **then**

                                *S*: = *S* ∪ {*u*};

                }

    **Return** *S* is a set of emerging influencers in *G*_*X*_.

**The complexity of Algorithm 2. Theorem 2:** The complexity of the algorithm 2 is:

$$O(n^{4}.m)$$ (30)

* Proof: There are two main stages in Algorithm 2:

+ Stage 1: Determine the group of lovers of brand *X*.

This stage is worked by Algorithm 1. Through Theorem 1, the complexity of Stage 1 is:

$$O(\mathrm{card}(L_{X}).n^{2}.m.\omega )$$ (25)

+ Stage 2: Determine the emerging influencers with the brand *X*. There are two main steps in this stage: Step 3 and Step 4.

- Step 3: Identify values of the influent metrics.
- Step 4: Detect the set of influencers.

At Step 3, the complexity for computing metrics are as follows:

○ Influent vector: IU(*u*): = (*Imp*(*u*), *Popularity*(*u*)) (1)

Because *Imp*(*u*) and *Popularity*(*u*) are determined based on each user’s collected data, the complexity of computing an influent vector is: *O*(*n*) (31)

○ Content creation score: *CC*_*X*_(*u*): = *PP*_*X*_(*u*) + log(*Q*(*u*)) (14)

The value of *Q*(*u*) is computed by the number of user *u*’s posts, *PP*_*X*_(*u*) is determined based on collected data of user *u*. Thus, the complexity of computing *CC*_*X*_(*u*) is: *O*(*n*^2^.*m*) (32)

○ By the Formula (28), the average of interactions based on the content creation for *u*’s posts related to the brand *X* in the time window δ, *AICC*_*u*_^*X*^(δ), is:

$$AICC_{u}^{X}(\delta )≔\frac{\sum_{\begin{array}{l} p\in u.ListPosts\\ p\mathrm{is}\;\mathrm{related}\;\mathrm{to}\;X \end{array}}SP_{p}^{X}(\delta )}{\mathrm{card}(u.ListPosts)}$$ (28)

$$\mathrm{where}\;SP_{p}^{X}(\delta )=\sum_{v\in IPCC_{p}^{X}(\delta )}\mathrm{card}(I_{p}^{v}(\delta ))$$ (27)

$$\begin{array}{l} I_{p}^{v}(\delta )=\{user\in U|(user,\pi_{user})\in U\times \mathrm{TIME},\\ \;user\in (p.\mathrm{Reaction}\cup p.Sh\cup p.Com),\\ \;user\neq v,\;\pi_{user}\in [p.\tau ,p.\tau +\delta ]\\ \;\} \end{array}$$ (2)

In which, π_*user*_ is the timestamp when the user reacts, shares, or comments on the post *p*.

For each post *p*, the complexity of (2) is: *O*(*n*^2^)

Because $\mathrm{card}(IPCC_{p}^{X}(\delta ))\leq n$, the complexity of (27) is: *O*(*n*^3^)

From Lemma, the complexity for determining the post *p* related to the brand *X* is:

$$O(\mathrm{card}(L_{X}).word(p))$$ (15)

Each user *u* has *m* posts. For each post of user *u*, we will check the relation between that post and the brand *X*, and estimate $SP_{p}^{X}(\delta )$ through the Formula (27). Thus, by the complexity of (27), we have the complexity of (28) for each user *u* as follows:

$$\begin{array}{l} O(m.\max(card(L_{X}).word(p),n^{3}))\\ =O(\max(card(L_{X}).word(p).m,n^{3}.m))\\ \approx O(\max(card(L_{X}).\omega .m,n^{3}.m))\\ \approx O(n^{3}.m) \end{array}$$ (33)

with *ω* is the average number of words for each post.

From (33), the complexity of computing the average of interactions based on posts related to the brand *X* in the time window δ is:

$$O(n^{4}.m)$$ (34)

From the formulas (31)(32)(34), the complexity of Stage 2 of Algorithm 2 is:

$$O(\max(n,n^{2}.m,n^{4}.m))=O(n^{4}.m)$$ (35)

+ Through the complexity of Stage 1 and Stage 2 as (25) and (35), the complexity of Algorithm 2 is as follows:

$$\begin{array}{l} O(\max(\mathrm{card}(L_{X}).n^{2}.m.\omega ,n^{4}.m))\\ =O(n^{4}.m)\;(q.e.d) \end{array}$$ (36)

## Testing and experimental results

Nowadays there already exist several companies that provide marketing management tools, which will be covered in more detail in the rest of this subsection, such as: Hiip [67], ViralWorks, [68]. However, due to business purposes, solution providers have never released details of their solutions or revealed detailed statistics. Hence, we aim to design a holistic solution to both publish to the community and empower brands through the entire process from selecting the appropriate influencers, using a more accurate marketing efficiency measurement tool to generating more sales. To demonstrate the effectiveness of this novel system, we compared the effectiveness of Influencer marketing campaigns in which Influencers are identified by our system with the results of actual Influencer marketing campaigns that the brands conducted before.

**Fig 3:**
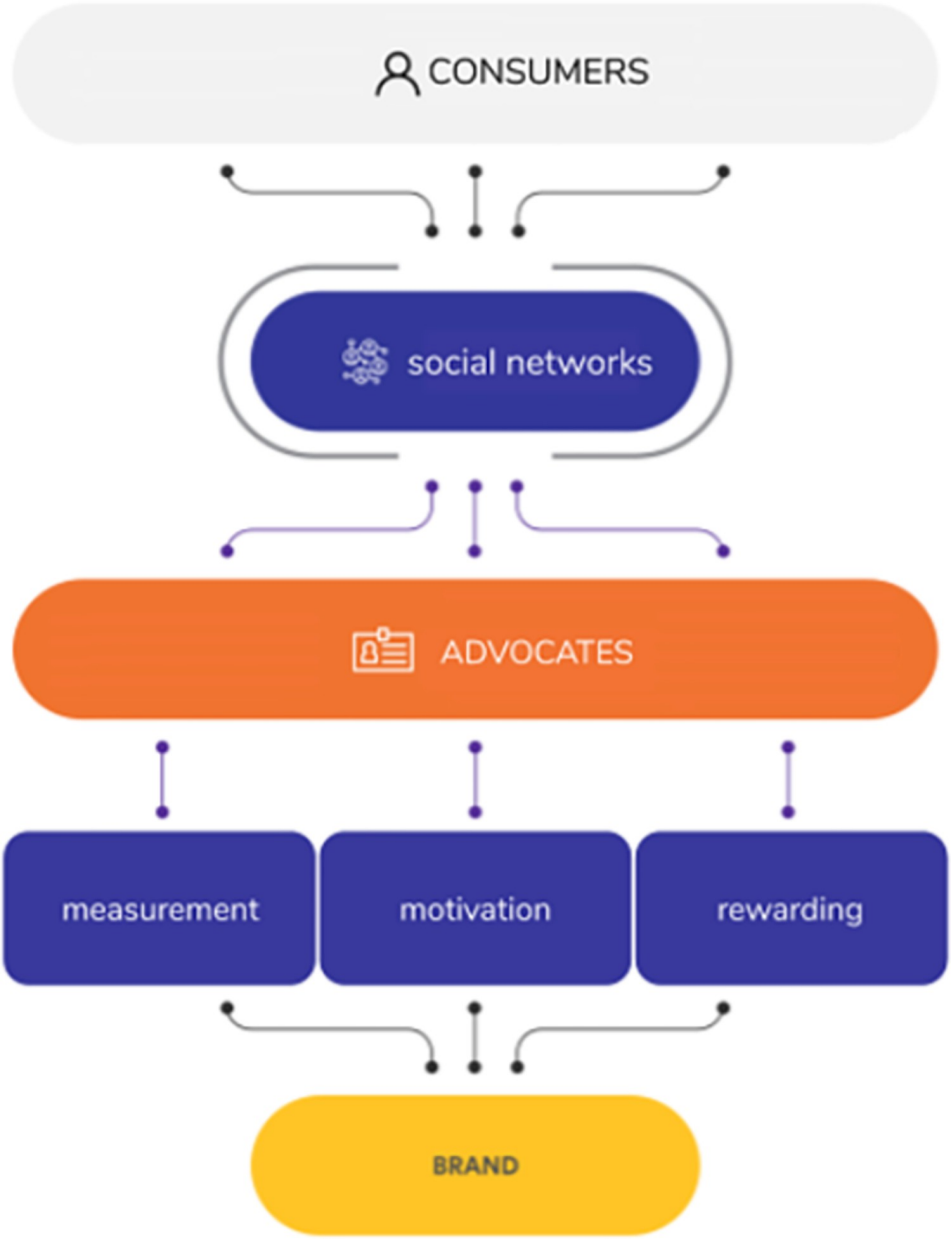
The work of the system for detecting of influencers.

Our proposed method has been used to detect the influencers of a brand. From the list of brand’s consumers, by computing their measure on the social network, the system uses the proposed measures to detect influencers to viral this brand. Those influencers will be the crucial factor in running an influencer marketing campaign for the brand. The work of the system is shown in Fig 3.

The method begins by putting together a database of social media users and their posts. A crawling engine will acquire those users from social media. An initial data set will be entered to improve the relevance of crawling users to a specific brand. These initial data might be a list of influencers from prior campaigns, hashtags, groups, or other information that the crawler can use to create the database. Simultaneously, a scoring engine will track the two metrics indicated above, including the amplification factors and the content creation score. These data will be reviewed by business users for their influencer campaign, and they will be regularly monitored and optimized. In addition to these engines, a front-end system for influencers is being developed with the goal of allowing businesses to use gamification to inspire and nurture them. Gamification’s use cases can simply be that the better and appealing posts/comments are, the more influencers can be rewarded. The system can also establish an affiliate connection to a company’s e-commerce platform, allowing influencers to be judged not just on their amplification and content production, but also on the income generated by customers who bought products after seeing them on social media.

The primary function of this system is to determine how influential people are on social networks, and then to assist businesses in increasing brand recognition and conversion by leveraging these scores through gamification. As a result, this application can be used for a variety of corporate purposes, such as a brand ambassador campaign, staff advocacy campaign, or a review-to-earn, share-to-earn strategy [69]. Influencer marketing appears to be most commonly used to increase brand awareness. However, from a commercial standpoint, the money generated by any marketing campaign is an important metric to track. This section demonstrates how the proposed strategy can be utilized for influencer commerce in addition to boosting awareness on social media. Influencer commerce is a new strategy that brands and marketers are employing to drive leads and sales. This strategy will alter how influencers generate money as well as provide additional options for businesses to make direct sales.

### Comparing with SNOL and SP approaches

The SNOL (Social Network Opinion Leaders) score is proposed in the study [70], which is an ensemble of those features using the adjustable parameters. These parameters are identified by using a fuzzy-based algorithm that follows work from [71]. In particular, the SNOL score in [70] was experimented on the dataset collected from Twitter. Since our experiment data is collected from Facebook, there are some efforts to transform the attributes to fit the specification of Facebook data. Firstly, the retweet action in Twitter is defined as the sharing one in Facebook. Secondly, a tweet in Twitter is also understood as a post in Facebook. The rest of features such as focus rate, activeness, authenticity, etc. remain the same meaning.

To detect influencer on Instagram, the work from [72] takes advantage of Social Network Analysis approach. Particularly, they study the spreading (SP) behavior on a structure of knowledge graph. By using the Linear Threshold Model, the algorithm calculates the proportion of nodes reached and the number of days required to reach the limit of the graph. The SP score is defined to detect and measure the influence score of a user by dividing the proportion of nodes reached to the number of days required.

We also demonstrate the algorithms to calculate the SNOL and SP scores based on our dataset. With SNOL approach, the opinion leaders, known as influencers, are detected by *k* dominant clusters out of *N* ones using the K-means algorithm on the features. Then, a SVM model is fitted to tune the adjustable parameters. The SNOL score is calculated on our dataset. Since it is calculated based on each topic, in this experiment, the SNOL score is averaged of all current topics of the dataset to get the final SNOL score. With SP approach, this algorithm in our structure of knowledge graph induced, and the SP score is calculated with the Linear Threshold Model. Those scores are compared to the proposed method by the cosine similarity with the baseline engagement score.

The dataset is collected from Facebook from 06-08-2018 to 06-09-2019. There were 18,949 users were crawled, and we removed 15,074 users who cannot collected any posts during the collected time. There are 9,225 remaining users with 312,130 posts and 112,180,524 interactions.

**Fig 4:**
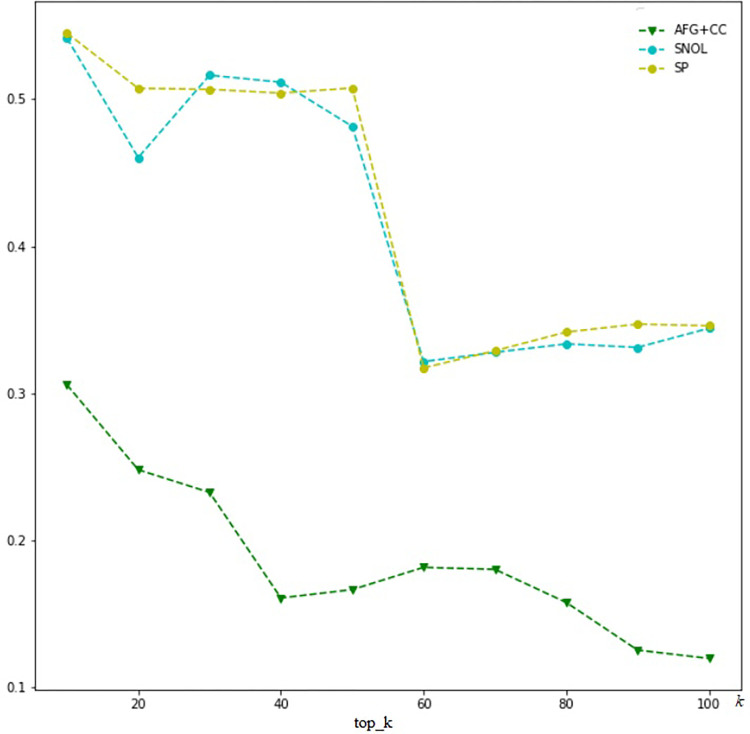
Similarity scores of AFG + CC, SNOL and SP approaches.

Fig 4 compares the similarity scores between the proposed method (called Amplification factors combine content creation score, AFG + CC), SNOL and SP approaches.

**Fig 5:**
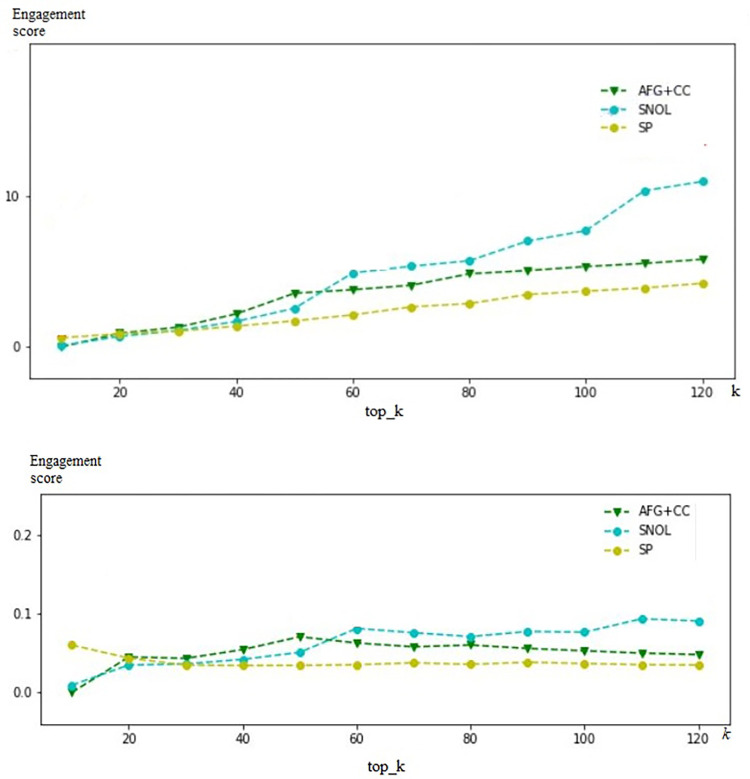
Comparing the engagement score of users using AFG+CC, SNOL and SP approaches. a. Total of engagement scores of users in top_k. b. The average of engagement score of users in top_k.

This figure shows that the results of the proposed method are different from other methods. Because, the AFG+CC approach focuses to detect micro-influencers for the brand, and other approaches tend to determine celebrities for it. However, the total engagement score of the proposed method is more effective than others when selecting a small group of users (*k* < 50) and better than the SP approach when expanding the group of users. Those results are shown in Fig 5.

### Application in a practical marketing campaign

This section presents the results when applying determined influencers in a practical marketing campaign. Because of the business secret, our customer’s brand is called the brand *X*, and the time window δ is six (06) days. A campaign of influencer marketing was done in February 2020, and it only considers Vietnamese users on Facebook. This campaign was separated into two phases:

- Phase 1: From Feb. 12–18, 2020. The customer used 31 micro-influencers for their brand *X*; our customer determined those influencers by themselves.
- Phase 2: From Feb. 18–23, 2020. The customer used ten micro-influencers who were determined by our measures combining the content creation score.

#### Determine influencers by AFG+CC approach

The determination of influencers for the product *X* in Phase 2 is processed by Algorithm 2.

**Fig 6:**
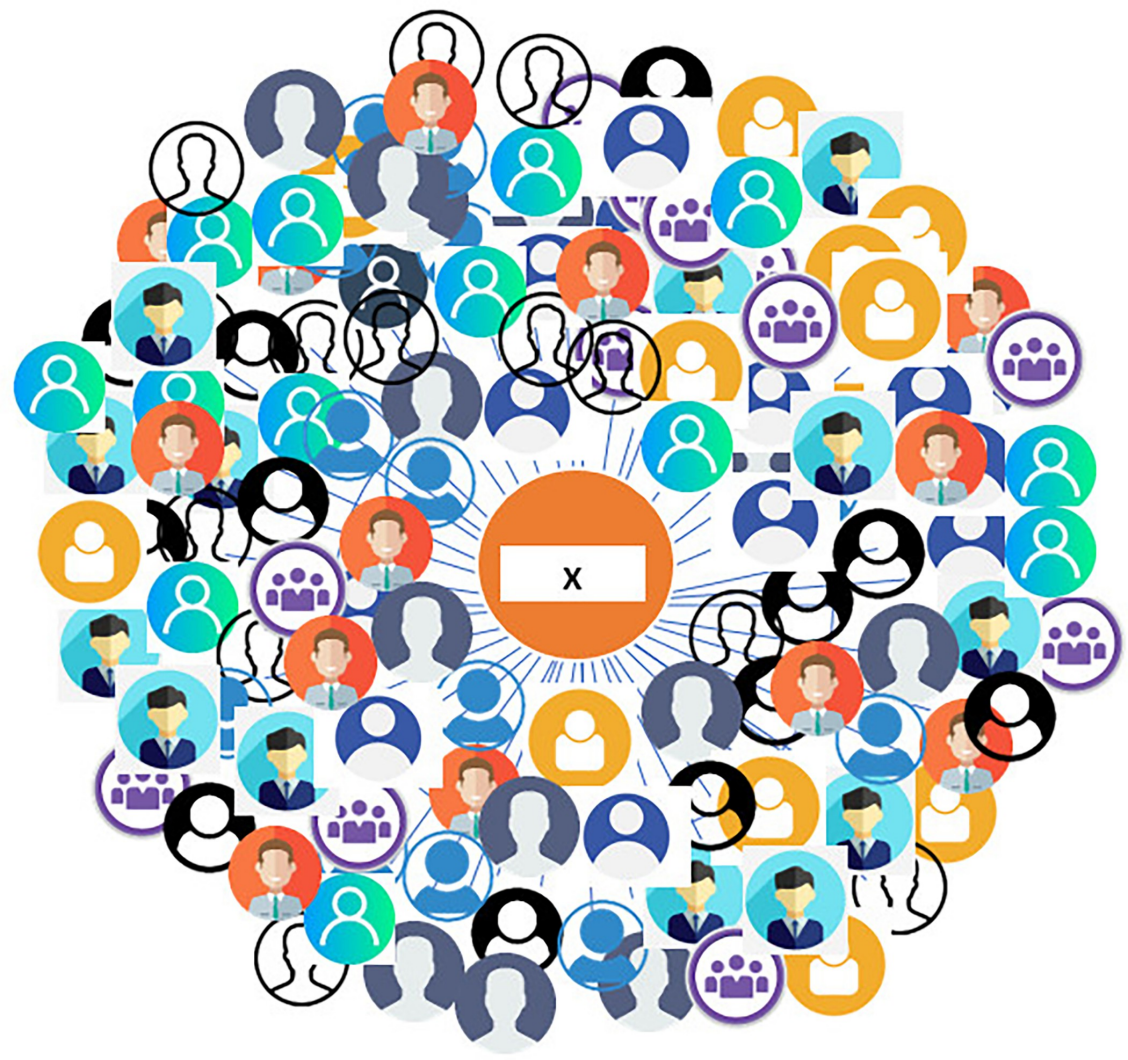
Sub-graph presents the homophily, including people interested in the brand *X*.

Stage 1: Using the information of *X*, a sub-graph representing a group of brand-lovers of *X* is shown in Fig 6:

Stage 2: Through this group, the emerging influencers for brand *X* in the time window δ = 6 days) are determined using the proposed measures. Using the opinions from the experts and managers in online marketing, the values of parameters in formulas were chosen as follows:

- The values of (α_1_, α_2_, α_3_), (β_1_, β_2_, β_3_), (γ_1_, γ_2_, γ_3_) in Table 2, and (α, β, γ) in Def. 3.4:

α_3_ = 0.75 α_2_ = 0.5 α_1_ = 0.25

β_3_ = 0.75 β_2_ = 0.5 β_1_ = 0.25

γ_3_ = 0.75 γ_2_ = 0.5 γ_1_ = 0.25

α = 0.5 β = 0.5 γ = 0.5

- The value of *μ* in Eq (29) is selected by 0.7, which means a user is a potential, influential user if he/she is more influential than 70% of members in the group *G*_*X*_.

**Table 2:** List of emerging influencers.

User ID	AFG + CC score
rLZaWQ3JqFYwAmA47oGY	16.03
JKxo9aAU83Bxsuli3Xdg	15.98
EWfLBNoBIt4zepQz14y3	14.21
1OqWFsKGILyNJReojcum	14.12
5JNxo5vXnHuFTbH5owfW	14.05
P7gfjj2bQN1hC8htFLjz	13.24
Y3o8jqbhZsqNvv8jSoW0	12.84
WLH8M8GLuYUVoq0cPrPh	12.36
3nqAvZEMVZ4PnKBQ3zNK	11.02
wx6UtNwXIsowltmFSgfO	10.91

The list of potential influencers is shown in Table 2. Ten users can become influencers for product *X* to run the influencer marketing campaign of our customers.

#### Experimental results

In the followed results, we compare the impact of information propagation and interactions with the post related to brand *X* in two phases. A marketing campaign’s effectiveness is evaluated based on the number of clicks on interactions, the conversion rate of clicks to orders, and the revenue.

**Fig 7:**
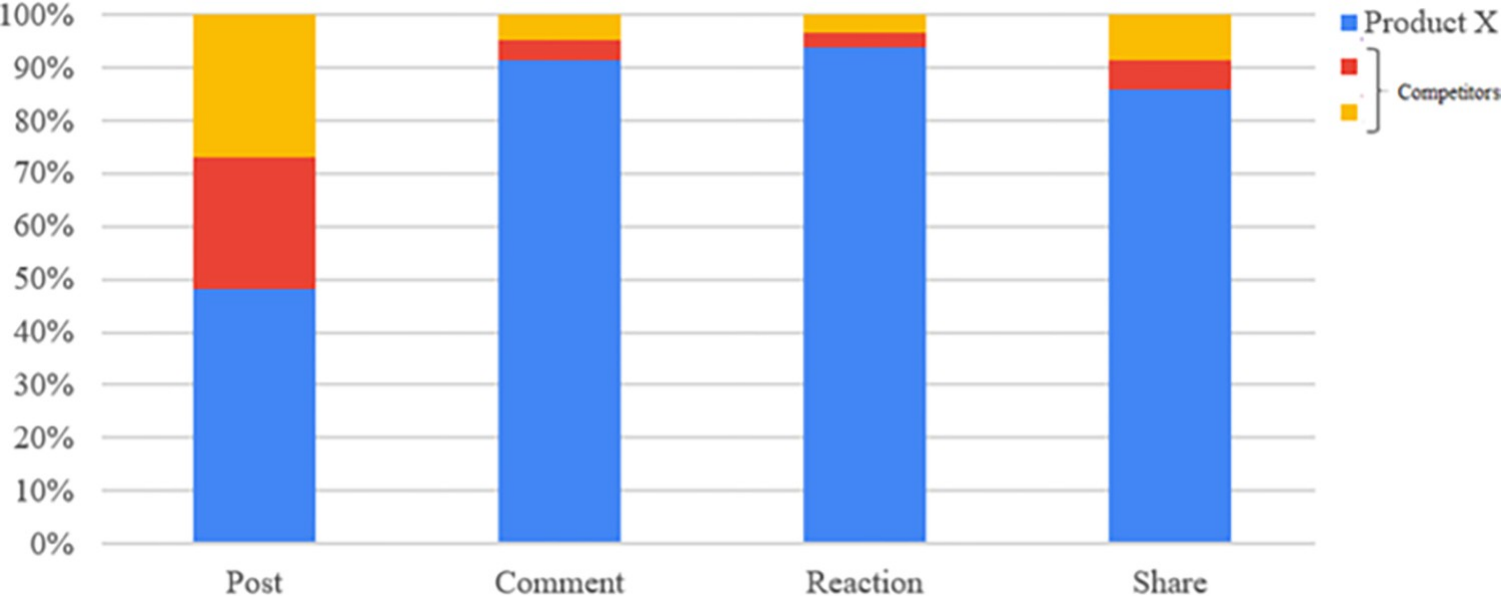
The voice of interactions between products from 13–23 February 2020.

Table 3 and Fig 7 compare the number of interactions on the brand *X* to other competitors in February 2020. The results show that product *X* being more interacted than others.

**Table 3:** Compare the interactions related to brand *X* and competitor products from 13–23 Feb. 2020.

Product	Post	Reaction	Comment	Share
Product *X*	41	2,366	11,323	193
Competitor 1	21	101	317	13
Competitor 2	23	123	426	19
Total	85	2,590	12,066	225

**Fig 8:**
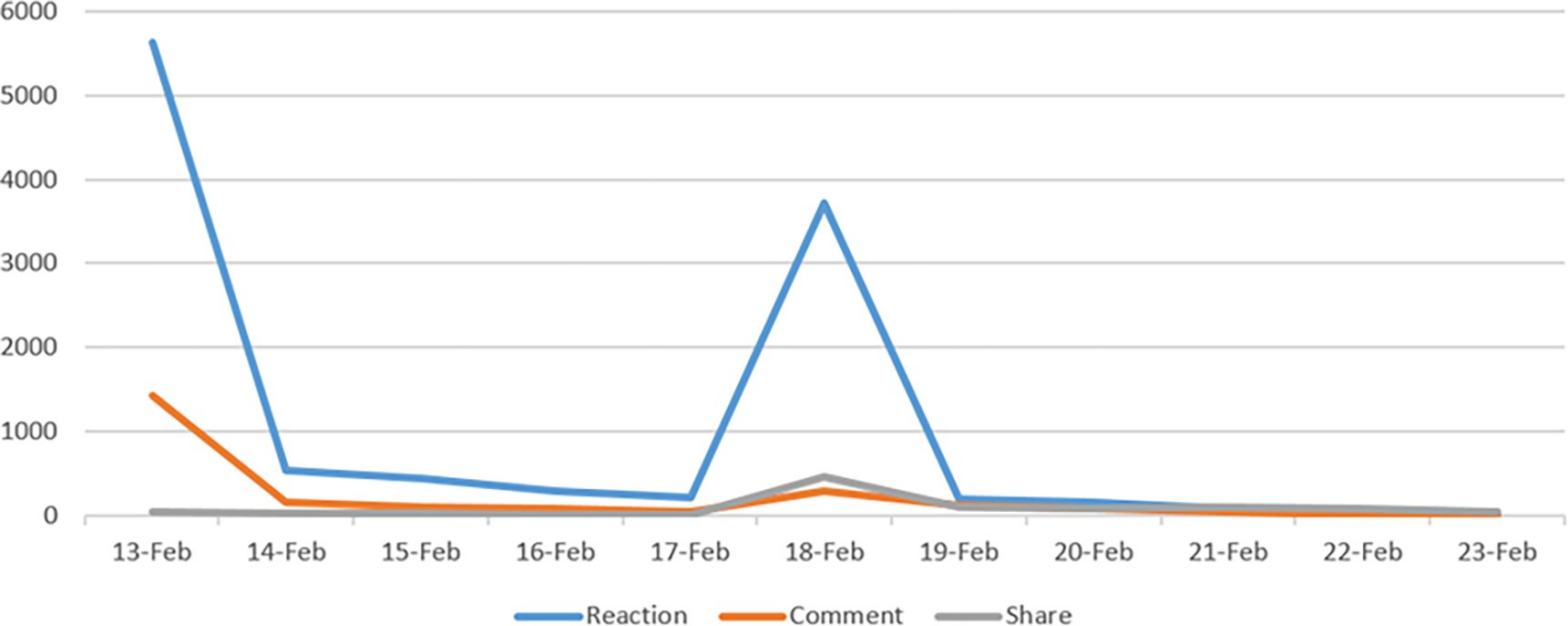
The interactions related to the brand *X* from 13–23 February 2020.

Table 4 and Fig 8 show the number of interactions related to the brand *X* in each phase of this influencer marketing campaign.

**Table 4:** Total of interactions that are related to the brand *X* in February 2020.

Phase	Post	Reaction	Comment	Share	Total
Phase 1	31	7,242	1,823	112	9,177
Phase 2	10	4,081	543	81	4,705

**Fig 9:**
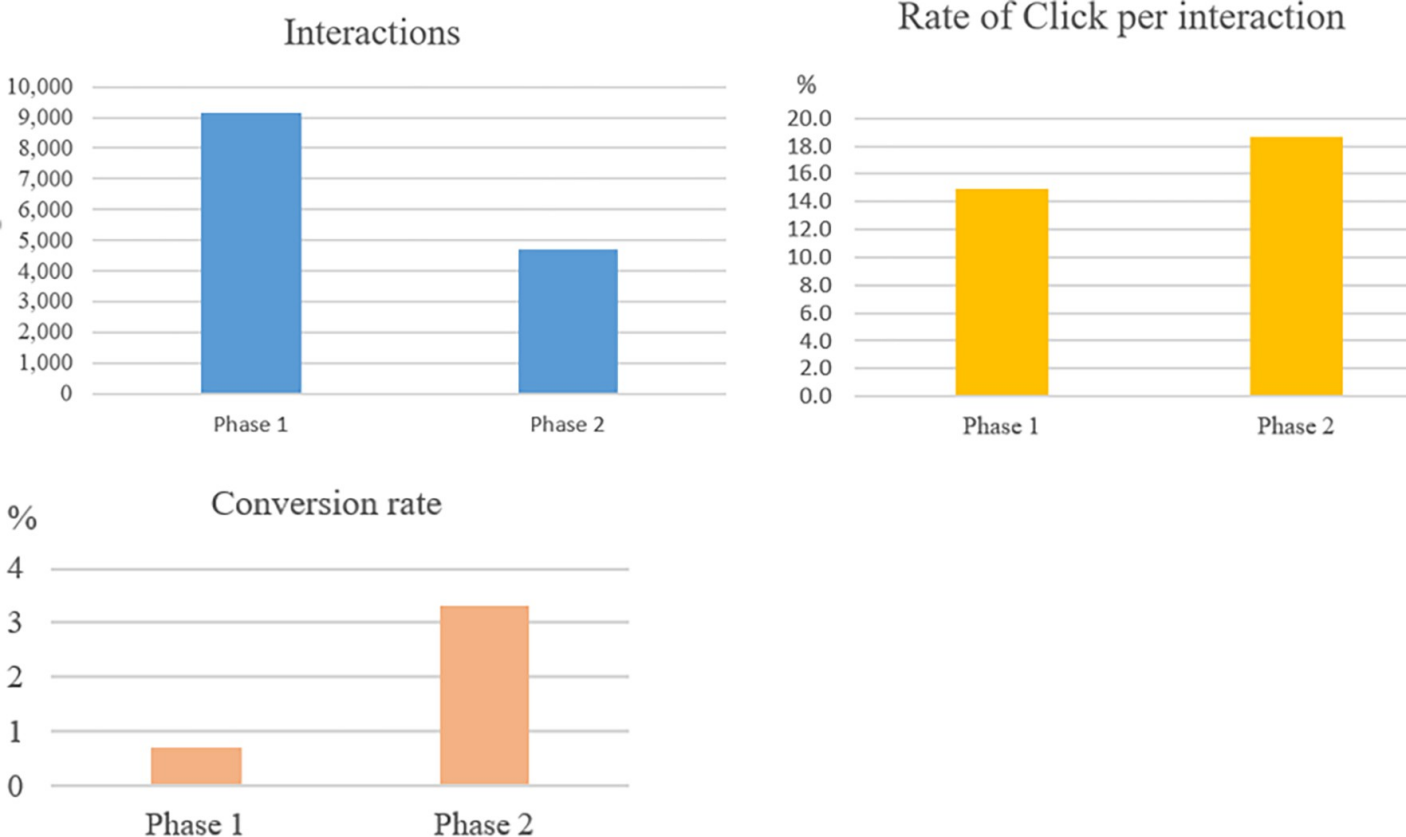
Compare the factors between the two phases.

In the influencer marketing campaign of the product *X*, Table 5 shows the number of interactions on the posts, and the numbers of clicks, orders in two phases. Fig 9 compares those values between two phases in this campaign.

**Table 5:** Comparison of factors between the two phases in the campaign.

Factors	Phase 1	Phase 2
Number of Posts	31	13
Number of Interactions	9,177	4,705
Number of Clicks	1,368	878
Number of Orders	9	29
Rate of clicks per interaction	15.1%	18.8%
Conversion rate ^1^	0.7%	3.3%

^1^ The rate of orders per click.

**Fig 10:**
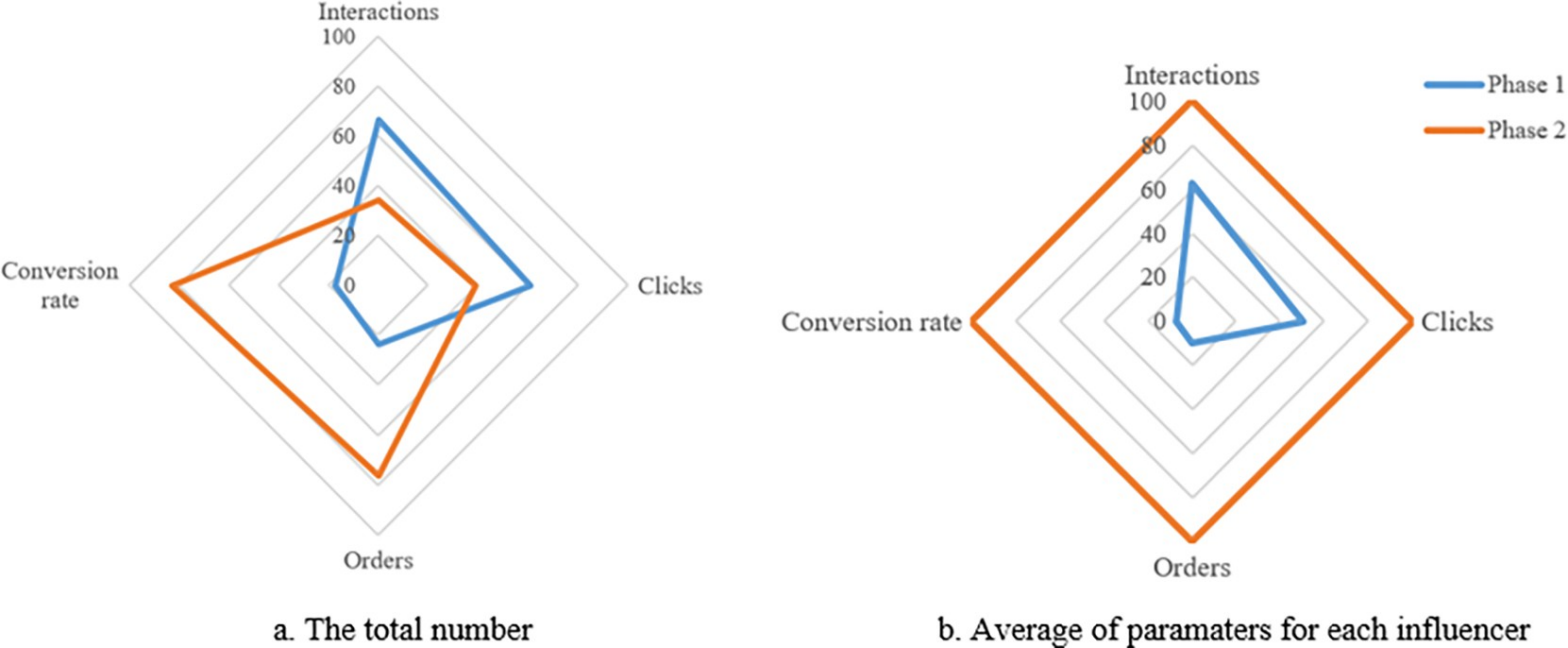
The total number of parameters and average of parameters for each influencer in two phases of the campaign.

Although phase 1 has several more interactions than phase 2, both the click per interaction and the conversion rate of phase 2 are better than phase 1. Hence, the revenue of phase 2 is higher than phase 1. In the practice, phase 2 gives more benefits than phase 1 for our customers. Fig 10 shows that the voice of the conversion rate and orders of phase 2 is more massive than phase 1. Besides, the average sale for each influencer in phase 2 is more significant than each influencer in phase 1 (Fig 10B). Thus, the result of phase 2 is more effective than phase 1.

Table 6 only analyzes comments which are interacted on the posts of the customer’s influencers by their sentiment in this campaign.

**Table 6:** Analysis the sentiment of comments in each phase of the campaign.

Phase	Positive	Negative	Neutral	Not Concerned	Total
Phase 1	1,481	45	251	46	1,823
Phase 2	457	13	65	8	543
^1^Rate of phase 1	81.2%	2.5%	13.8%	2.5%	
^2^Rate of phase 2	84.2%	2.4%	11.9%	1.5%	

^1^ The rate between each kind of sentiment and total comments in phase 1.

^2^ The rate between each kind of sentiment and total comments in phase 2.

Table 6 shows that the rate of positive comments in phase 2 is higher than in phase 1. The rate of negative comments is similar. Because the influencers in phase 2 tend to the brand *X*, their engaged audiences’ interest also tends to the brand *X*; thus, the rates of not-concerned comments and neutral comments in phase 2 are lower than in phase 1. The post-contents in phase 2 are better and more attractive, getting more positive feedback from audiences.

Through the above results, the proposed method is helpful to identify potential influencers for a determined brand. It brings the efficiency of the conversion rate and the revenue in the influencer marketing campaign. After running the experimental campaign in the real world, our customers also give good feedback for our method.

## Discussions

The proposed effectively searches the influencers of a product/brand on the Vietnamese social network. Our method is the combination of the measures of information propagation and content creation to determine emerging influencers. This approach is built by the measure of passion point and the technique of sentiment analysis. Moreover, this method can be applied to many products or brands that can be approached on an online social network. When applying this method in another field, we only need to build the corpus of that field for crawling data in that field. The collected data is about the community of users and their activities on the social network. The proposed method has been used to build a system to manage influencer marketing campaigns on the social network [73].

Our method can work well on Facebook; however, some information propagation factors have to change appropriately when applying a social network platform to another social network platform. For example, on Twitter, the point of reactions of a post, *react_point*(*p*), needs to be changed when applied. Sentiment analysis is worked based on the corpus of a language. Hence, when applying the proposed method in another language, the corpus for that language needs to be constructed.

## Conclusion and future work

In this paper, based on the SNet model, the amplification factors of a user are determined. They have used a method for estimating the user’s information propagation, which has been improved from [54]. This method is built by using the social pulse for a post in the time window δ. Besides, the method for estimating the user’s content creation score on social networks is also proposed. This score is determined by combining the passion point and analysis of the post’s content attraction. The passion point is evaluated by the sentiment score of posts and user’s activity. The post’s content is analyzed by using sentiment lexicons. The content creation score measures the interest of a post to attract interactions from audiences. We have used the measures to evaluate content creation and information propagation; the method for detecting potential influencers has been proposed. This method can detect influencers impacting other users on social networks with a brand or a product. Moreover, those determined influencers also can create engaging posts for their audience. Those influencers are emerging to run the influencer marketing campaign for that brand/product.

In the experiment, the proposed method, called AFG + CC, is compared with other approaches, SNOL and SP. The results show that the proposed method detecting micro-influencers for the brand, and other approaches tend to determine celebrities for one. However, the total engagement score of the proposed method is more effective than others when selecting a small group of users (*k* < 50) and better than the SP approach when expanding the group of users. Moreover, the AFG + CC method is applied to run a real-world influencer marketing campaign. This experiment shows that the influencers, which are detected by our method, are more effective than others. They bring the efficiency of the conversion rate and the revenue in the influencer marketing campaign.

In the future, our method will be tested on other platforms of social networks, such as Twitter [74], Zalo [75]. Moreover, the measure of content creation will be improved to become a general method for evaluating the post’s content. The improved method can be applied to increase the effectiveness of a content marketing campaign. Although the SNet model can be applied to represent the structure of social networks, some techniques will also be studied more to process many kinds of collected data, such as images and clips. Those improvements can be applied in other media platforms of social networks, such as Instagram [76], Tiktok [77].

The recognition of consumer behaviors is vital to approach target customers. In further research, the method for determining the changes in behaviors has been studied. This method can combine with content creation and information propagation measures to determine influence diffusion on the social network [78] and establish an effective online marketing strategy for a specific commercial brand [20, 79].
